# Solid-state NMR investigation of the involvement of the P2 region in tau amyloid fibrils

**DOI:** 10.1038/s41598-020-78161-0

**Published:** 2020-12-03

**Authors:** Adriana Savastano, Garima Jaipuria, Loren Andreas, Eckhard Mandelkow, Markus Zweckstetter

**Affiliations:** 1 German Center for Neurodegenerative Diseases (DZNE), Von-Siebold-Str. 3a, 37075 Göttingen, Germany; 2 Max Planck Institute for Biophysical Chemistry, Am Faßberg 11, 37077 Göttingen, Germany; 3 German Center for Neurodegenerative Diseases (DZNE), Venusberg-Campus 1, 53127 Bonn, Germany; 4 CAESAR Research Center, Ludwig-Erhard-Allee 2, 53175 Bonn, Germany

**Keywords:** Biophysics, Intrinsically disordered proteins, NMR spectroscopy, Proteins, NMR spectroscopy

## Abstract

The aggregation of hyperphosphorylated tau into amyloid fibrils is closely linked to the progression of Alzheimer’s disease. To gain insight into the link between amyloid structure and disease, the three-dimensional structure of tau fibrils has been studied using solid-state NMR (ssNMR) and cryogenic electron microscopy (cryo-EM). The proline-rich region of tau remains poorly defined in the context of tau amyloid structures, despite the clustering of several phosphorylation sites, which have been associated with Alzheimer’s disease. In order to gain insight into the contribution of the proline-rich region P2 of tau to amyloid fibrils, we studied in vitro aggregated amyloid fibrils of tau constructs, which contain both the proline-rich region P2 and the pseudo-repeats. Using ssNMR we show that the sequence $^{225}{\text {KVAVVRT}}^{231}$, the most hydrophobic patch within the P2 region, loses its flexibility upon formation of amyloid fibrils. The data suggest a contribution of the P2 region to tau amyloid fibril formation, which might account for some of the unassigned electron density in cryo-EM studies of tau fibrils and could be modulated by tau phosphorylation at the disease-associated AT180 epitope T231/S235.

## Introduction

The intrinsically disordered protein tau is associated with the onset of Alzheimer’s Disease (AD) and other neurodegenerative diseases, also known as tauopathies^1–4^. These diseases see the common event of pathological aggregation of tau into amyloid fibrils, which further accumulate in different forms of aggregates, e.g. Neurofibrillary tangles (NFTs) and Neuropil threads (NTs), in the neurons^1,3^. Tau belongs to the family of the microtubule-associated proteins (MAPs)^5,6^ and it is mainly expressed in the axons of neuronal cells, where it interacts with microtubule filaments^7–9^. Six isoforms, differing in sequence composition, are expressed as the result of alternative splicing of exons 2 and 3 of the MAPT gene^10^. The longest tau isoform, called htau40, is a protein of 45.9 kDa comprising two insertions of 29 amino acids each (N1 and N2), a proline-rich region, which can be subdivided into a P1 and a P2 domain, five pseudo-repeats (from R1 to R4 and ${\text {R}}^\prime$) and a C-terminal tail^5,11^. Because of its link to neurodegenerative diseases, tau and the formation of tau fibrils have been studied over the years with the aim to understand the molecular mechanisms underlying the aggregation process.

Tau fibrils are characterized by a solvent inaccessible rigid core and a fuzzy coat, which can be removed by protein digestion^12^. Several studies have been directed towards the identification of the protein domains responsible for its aggregation^13–17^. Tau is an intrinsically disordered protein with high solubility and a low aggregation propensity in vitro^18^. In vitro aggregation of tau has been traditionally achieved using polyanions like heparin^19,20^ and arachidonic acid^21^, which, due to their negative charges, promote tau aggregation by neutralization of the positive charges on tau. Biochemical studies could define that the rigid core of the fibrils is composed of the pseudo-repeat region and that the hexapeptide $^{306}{\text {VQIVYK}}^{311}$ at the beginning of the R3 domain represents the minimal sequence able to self-assemble into fibrils^17,22^. Studies with small tau constructs evidenced a similar role for the hexapeptides $^{275}{\text {VQIINK}}^{280}$, in the R2 domain, to form *bona fide* fibrils^21,23^. NMR analysis of tau secondary structure in solution reported residual $\beta$-sheet structure for the two hexapeptides^15,24–26^: these were hypothesized to provide hydrophobic contributions that in pathological conditions can enhance protein aggregation. The fuzzy coat, composed by the N-terminal domain, the proline-rich region and the C-terminal domain, maintains a higher degree of flexibility in the fibrils^14,27,28^; nevertheless transient contacts between the proline-rich region and the core of the fibrils could be detected using NMR spectroscopy^14^ supporting its possible roles in tau aggregation^29^. Solid-state NMR (ssNMR) spectroscopy, electron microscopy (EM), X-ray diffraction, electron paramagnetic resonance (EPR) and other biophysical techniques have been employed to gain insight into the structure of tau fibrils^13–16,27,30–39^. One of the challenging aspects that has made the structural characterization difficult is the observation that tau fibrils are often heterogeneous^40,41^.

Recently three-dimensional structures for fibrils of tau in AD and Pick’s disease (PiD) have been determined using cryogenic EM (cryo-EM)^30,31^. Tau fibrils purified from the brain of an AD patient exhibited two morphologies, paired helical filaments (PHFs) and straight filaments (SFs). Both types of fibrils appeared to be composed by two C-shaped protofilaments comprising the R3 and R4 domains^31^. The hexapeptide $^{306}{\text {VQIVYK}}^{311}$ is engaged in the formation of a cross-$\beta$ sheet structure with residues in the ${\text {R}}^\prime$ region, while the inter-repeat PGGG motif at the end of the R3 domain forms a turn, which gives the C-shape to the structure. The different ways of interaction between the identical PGGG motifs in the PHFs (symmetrical) and in the SFs (asymmetrical) are the source of the two distinct polymorphs. The resolution quality up-stream to the R3 repeat and downstream to the ${\text {R}}^\prime$ repeat prevented insights into other parts of the protein. Indeed, additional electron density at the N- and C-termini of the cross-$\beta$ structure suggested that additional amino acids from the R1/R2 and ${\text {R}}^\prime$ pseudo-repeats, respectively, could contribute to the structure of tau fibrils. On the other hand, the R2 domain was not considered part of the core because of its cleavage by pronase. Although there is evidence supporting contacts with the core of the fibrils^14^, the cryo-EM analysis did not provide information about the structural properties of the proline-rich region^31^.

Fibrils purified from the brain of a PiD patient where composed of the tau 3R isoform, which lacks the R2 domain. Two morphologies defined as Narrow Pick filaments (NPFs) and Wide Pick filaments (WPFs) could be distinguished^30^. The NPFs are composed of a single protofilament, whose core is composed by the R1, R3 and R4 repeats, and adopts a hairpin-like shape *via* hydrophobic interactions between the strands^30^. The WPFs are composed of two NPFs associated together. In PiD, tau isoforms containing three pseudo-repeats are the most expressed^1^, therefore the R2 domain was not modelled in the cryo-EM structure.

Other cryo-EM three-dimensional structures, in this case of heparin-induced in vitro tau fibrils, have also been characterized^42^. The resulting structures differed from AD and PiD fibrils structures by inclusion of the R2 domain in the core of the fibrils, which was not observed in the three-dimensional structures reported by Fitzpatrick et al. and by Falcon et al.^30,31^. In any of the structures resolved by cryo-EM, only the pseudo-repeat regions could be characterized, but no structural information was accessible for other regions of the tau sequence. In particular, the proline-rich region was not observed, leaving open questions regarding the contribution of this domain to the formation and structure of tau fibrils.

The relevance of the proline-rich region in pathological aspects of tau is supported by the presence of many phosphorylated epitopes for monoclonal antibodies, e.g. AT180, AT100, AT80 and PHF1, which recognize hyperphosphorylated disease-associated forms of tau^43–47^. The proline-rich region is also rich in serine and threonine residues, which make it a target for many kinases involved in the pathological hyperphosphorylation of tau^1,48^. In addition, the proline-rich region plays an important role for the interaction of tau with tubulin^7,49^ and is involved in signaling pathways: the proline-rich motifs (PRM, i.e. PP motif) within the sequence are recognized by proteins containing proline-rich motifs binding modules (PRM-binding modules) such as the SH3 domain^50^.

Here we addressed the involvement of the proline-rich region in the pathological aggregation of tau. We designed simplistic models of tau, namely the two polypeptides P2R2 and P2R3, in order to better understand structural properties of the P2 domain in tau amyloid fibrils. The heparin-induced in vitro fibrils obtained from these two polypeptides, together with those obtained from the tau construct K32, comprising the proline-rich and the pseudo-repeat regions, have been studied using aggregation assays and ssNMR spectroscopy. The combined data provide evidences for the recruitment of the proline-rich region P2 to the core of tau fibrils.

## Results

### Solution-state NMR of K32

**Figure 1:**
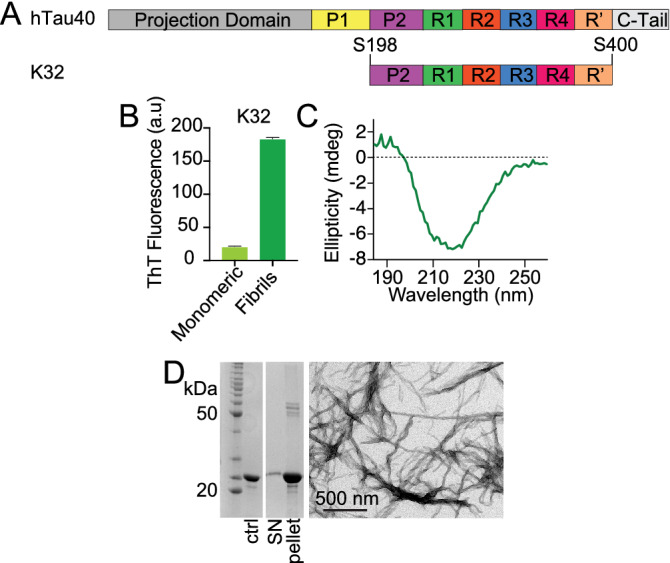
Aggregation of tau’s microtubule-binding domain into amyloid fibrils. (**A**) Domain organization of htau40 and the K32 construct. (**B**) ThT fluorescence intensities of the fibrils obtained from the aggregation of K32 (dark green) in presence of heparin. The ThT intensity for K32 in the monomeric form (light green) was used as control. Error bars indicate the standard deviation over three replicates. (**C**) CD spectrum of fibrils of K32: a minimum at 220 nm for $\beta$-sheet secondary structure was observed. (**D**) Left panel, SDS-PAGE gel of the fibrils of K32 after centrifugation (ctrl, negative control containing only K32 in the monomeric form; SN, supernatant removed after centrifugation); the full-length gel is presented in Supplementary Figure S8. Right panel, electron micrograph of the K32 fibril sample, for which ThT fluorescence measurements and SDS-PAGE gel electrophoresis were performed, scale bar 500 nm.

The K32 construct comprises the P2 domain and the pseudo-repeat region (from R1 to R4, including the flanking ${\text {R}}^\prime$ domain) of full-length htau40 (Fig. 1A)^7^. In order to obtain the backbone and side-chain assignment, NMR 2D and 3D experiments were performed on $200 \, \upmu \text {M}$ K32 in 50 mM sodium phosphate buffer at pH 6.8. The $^{1}\text {H}$-$^{15} \text {N}$ HSQC spectrum of K32 displayed little chemical shift dispersion, as previously reported^15,25^ (Supplementary Fig. S1A online). The resonance assignment of the 2D $^{1}\text {H}$-$^{15}\text {N}$ HSQC (Supplementary Fig. S1A) was validated using the assignment previously reported^25^. For the assignment of the C$\alpha$ and C$\beta$ resonances in the $^{1}\text {H}$-$^{13}\text {C}$ HSQC spectrum (Supplementary Fig. S1B), 3D H(CC)(CO)NH-TOCSY and (H)CC (CO)NH-TOCSY experiments were performed.

### In vitro aggregation of K32 into amyloid fibrils

Being an IDP, tau shows little tendency to aggregate in vitro. Polyanions, e.g. heparin, induce in vitro tau aggregation by neutralization of the positive charges and thus allow the establishment of hydrophobic interactions between residues from different tau molecules^15,20^. Four independent aggregation assays were performed using $100 \, \upmu \text {M}$ of K32 in 25 mM Tris-HCl buffer, at pH 7.4 to give a ssNMR sample. Heparin (Mr 2 × 10^4^ g/mol, Carl Roth, Germany) was added to each reaction mixture to a final concentration of $25 \, \upmu \text {M}$ (K32-to-heparin molar ratio 4:1). After 3 days of incubation at 37 °C to ensure completeness of the reaction, the formation of fibrils was confirmed by measurement of the ThT fluorescence (Fig. 1B, dark green bar). A reaction mixture containing only the monomeric form of the protein, and no heparin, was also incubated in the same conditions, in order to provide a negative control (Fig. 1B, light green bar). To quantify the amount of formed fibrils, the samples were ultracentrifuged, followed by separation of the pellet from the supernatant. Each fraction was subsequently loaded onto an SDS-PAGE gel to check the protein composition. According to the gel electrophoresis run, $\sim \, 90\%$ of the protein was present in the pellet, and $\sim \, 10 \%$ remained in the supernatant (Fig. 1D, left panel; the full-length gel is presented in Supplementary Figure S8). The pellet retrieved after ultracentrifugation was resuspended in deionized water and used for acquisition of a CD spectrum. The CD spectrum showed a minimum at $\sim \, 220 \, \text {nm}$, indicative of $\beta$-sheet structure (Fig. 1C and Supplementary Table S2). The presence of fibrils in the pellet was further confirmed by the observation of twisted fibrils by electron microscopy (Fig. 1D, right panel).

### Probing the rigid core of K32 fibrils

**Figure 2:**
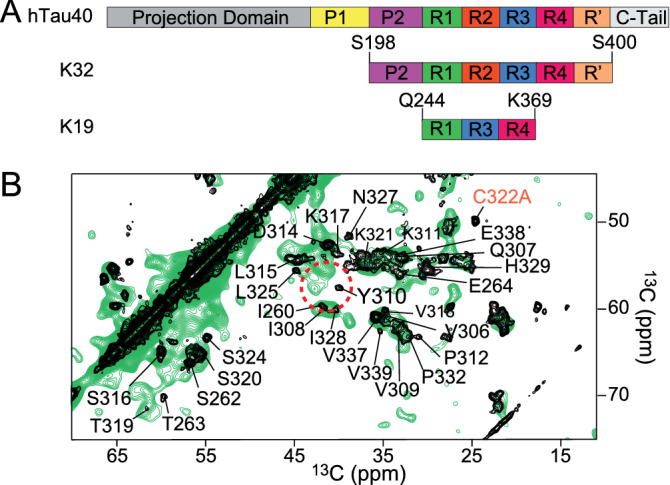
Solid-state NMR spectroscopy of the rigid core of K32 fibrils. (**A**) Domain organization of htau40 together with those of K32 and K19. (**B**) Superposition of the PDSD spectra of K32 (green) and K19 (black) fibrils, the latter one taken from Xiang et al.^40^; indicated is the assignment of K19 fibrils, reported by Xiang et al. as well^40^.

In order to understand the involvement of the tau proline-rich region P2 in the structure of tau fibrils, Proton Driven Spin diffusion (PDSD) experiments were performed on uniformly $^{15}\text {N}/^{13}\text {C}$-labeled K32 fibrils using 20 ms mixing time. Prior to the measurement, the fibrils had been ultracentrifuged (to remove residual monomeric protein) and were packed into a 1.3 mm magic angle spinning (MAS) rotor. The resulting spectrum (Supplementary Fig. S2) displayed signal overlap and few isolated cross peaks. Using the chemical shift values for amino acids in $\beta$-sheet conformation^51^, the residue type could be identified for some cross peaks, e.g. serine, threonine and proline residues, but no specific assignment could be performed (Supplementary Fig. S2). To tentatively assign some of the cross peaks, the spectrum obtained for the fibrils of the 3-repeat tau construct K19 was superimposed on the spectrum of K32 fibrils (Fig. 2B). The resonances assignment of the spectrum of K19 was previously reported by Xiang and collaborators^40^.

The K19 sequence contains the R1, R3 and R4 pseudo-repeats of tau (Fig. 2A), while the native cysteine residue in R3, C322, had been mutated to alanine in order to avoid the formation of inter-molecular disulfide bridges. This mutation is not present in the sequence of the K32 construct, thus the cross peak of C322A, which in K19 appears separated from the rest of the resonances, was not matching with any of the cross peaks in the PDSD spectrum of K32 (Fig. 2B). In Fig. 2B is shown that a larger number of cross peaks is visible in the 2D PDSD spectrum of K32 when compared to that of K19. In particular, more C$\alpha$/C$\beta$ cross peaks are present in the threonine and serine region of the K32 spectrum. The higher number of cross peaks in these regions is consistent with the relative abundance of these amino acid types: K32 contains 12 threonine and 22 serine residues, while K19 only contains 4 threonine and 8 serine residues. Because only rigid residues are detected in the PDSD spectrum, the comparison shown in Fig. 2B suggests that the K32 fibrils contain more cross-$\beta$ structure when compared to K19 fibrils. For several residues belonging to the R1 and R3 domains, cross peaks between the two spectra matched (Fig. 2B). For example, the cross peaks assigned to S262 (R1) and S316/S320 (R3) in K19 matched with cross peaks in the spectrum of K32. A similar observation was made for the cross peaks assigned to I260 (R1), as well as I308 and I328 (R3). The comparison suggests that residues belonging to the R1 and R3 pseudo-repeats are rigidified upon fibrillization of both K19 and K32.

K19 contains a single tyrosine, Y310, which is located in the R3 domain. In the 2D PDSD spectrum of K19, the C$\alpha$/C$\beta$ cross peak of Y310 is well separated from other signals, as shown in Fig. 2B. For K32, two cross peaks appeared in the spectrum in the same region, but could not be unambiguously assigned since two tyrosine residues, Y310 (in R3) and Y394 (in ${\text {R}}^\prime$), are present in the K32 sequence. In addition, the two potential tyrosine cross peaks could originate from a single tyrosine in two different fibril conformations. Nevertheless, the strong signal overlap in the K32 spectrum suggested that a different approach was needed to better understand to which extent the proline-rich region contributes to the structure of tau fibrils.

**Figure 3:**
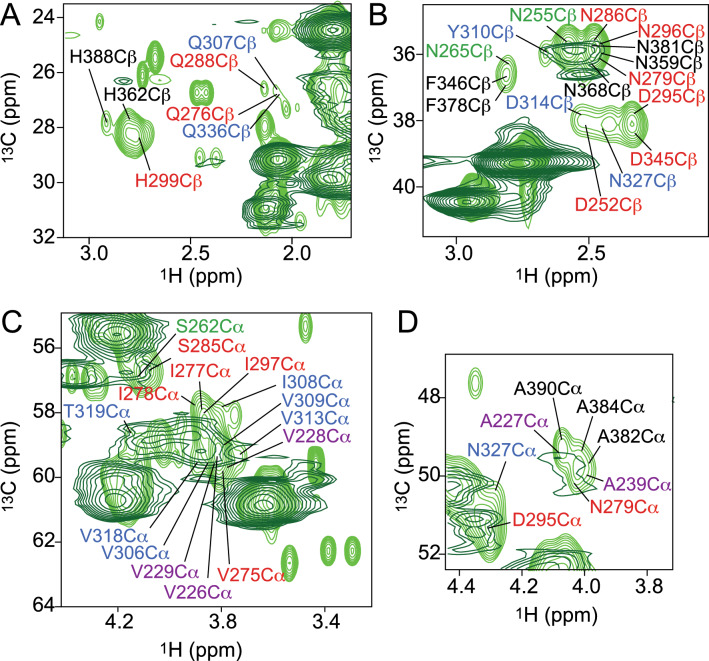
Superposition of the solution state $^{1}\text {H}$-$^{13}\text {C}$ HSQC spectrum (light green) and the ssNMR INEPT-based $^{1}\text {H}$-$^{13}\text {C}$ correlation spectrum (dark green) of monomeric and fibrillized K32, respectively. (**A**,**B**) Portions of the C$\beta$ region of the spectra revealing disappearing residues that belong to the pseudo-repeats R1 (green labels), R2 (red labels), R3 (blue labels), R4 and ${\text {R}}^\prime$ (black labels). (**C**,**D**) Portions of the C$\alpha$ region showing residues belonging to the R1, the R2, the R3, R4 and ${\text {R}}^\prime$ that experience signal intensity attenuation. A227 and A239 from the P2 domain (purple labels) are also affected (**D**).

### Detection of the flexible regions in K32 fibrils

Approaching the issue from a different perspective, a 2D INEPT-based $^{1}\text {H}$-$^{13}\text {C}$ correlation experiment was recorded on the sample of K32 fibrils. This type of experiment allows the detection of the flexible regions in a solid sample based on their different mobility^13^. In the transition from random coil to $\beta$-sheet conformation, the amino acid residues included in the fibril core lose flexibility and become invisible in an INEPT-based $^{1}\text {H}$-$^{13}\text {C}$ correlation experiment. Thus, the ssNMR INEPT and the solution state $^{1}\text {H}$-$^{13} \text {C}$ HSQC spectra were superimposed, with the purpose to reveal the tau residues that are not part of the rigid core of K32 fibrils. Upon superposition of the spectra, the cross peaks corresponding to the C$\alpha$/C$\beta$ resonances of proline, glycine, threonine and serine residues matched (Supplementary Fig. S3). Most of these residues are located in the proline-rich region, which is considered to be part of the fuzzy coat of the fibrils^14^. Thus, their visibility in the INEPT spectrum could be explained by their higher degree of mobility. The analysis of the domains considered to be in the rigid core of the fibrils was complicated by the signal overlap in the ssNMR INEPT spectrum, as well as the clustering of the C$\alpha$/C$\beta$ resonances in the $^{1}\text {H}$-$^{13}\text {C}$ HSQC (Supplementary Figs. S1, S3).

In the C$\alpha$ region of the INEPT spectrum, the signal intensities of the residues in the two hexapeptides $^{275}{\text {VQIINK}}^{280}$ and $^{306}{\text {VQIVYK}}^{311}$ in the R2 and R3 domains, respectively, were attenuated (Fig. 3C, residues in red for R2 and in blue for R3). In the C$\beta$ region of the spectrum, more isolated cross peaks, e.g. those of N265, Q276 and H299 located in the pseudo-repeat R1, D314, N327 and Q336 in R3, as well as H338, F346, D348, Q351 and H362 in R4, were broadened beyond detection in the ssNMR INEPT spectrum (Fig. 3A,B). Consistent with the PDSD spectrum, this suggested that the R1, R3 and R4 pseudo-repeats are included in the rigid core of K32 fibrils. Regarding the P2 domain and the $^{225}{\text {KVAVVRT}}^{231}$ sequence, signal intensity loss was observed for V226, A227, V228 and V229 (Fig. 3C,D), although the cross peaks did not completely disappear. Thus, signal overlap or a partial recruitmentt of the P2 domain to the core of K32 fibrils is likely.

### Preparation of the P2R2 and the P2R3 polypeptides and the characterization of their intrinsically disordered properties

**Figure 4:**
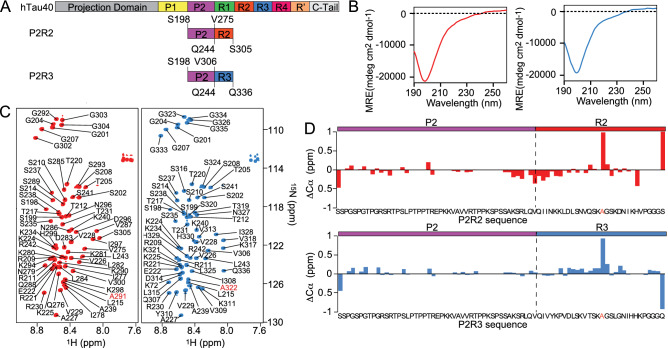
Intrinsically disordered properties of the P2R2 and P2R3 polypeptides. (**A**) Domain organization of the polypeptides P2R2 and P2R3 together with that of full-length htau40. (**B**) Circular dichroism spectra of $50 \, \upmu \text {M}$ P2R2 (red) and P2R3 (blue) in the monomeric form: a minimum at 200 nm indicative of random coil conformation is displayed in both spectra. (**C**) $^{1}\text {H}$-$^{15}\text {N}$ HSQC spectra of P2R2 (red) and P2R3 (blue) with the respective resonance assignments. (**D**) The C$\alpha$ chemical difference ($\Delta$C$\alpha$) calculated for htau40 and either P2R2 (upper panel, red) or P2R3 (lower panel, blue). The strongest chemical shift perturbation occurred in the regions surrounding the mutation C291A and C322A in P2R2 and P2R3, respectively.

To decrease signal overlap and thus obtain further insight into a potential contribution of the P2 region to the rigid core of tau fibrils, we prepared the polypeptides P2R2 and P2R3. These are simplified models of tau, because they combine one domain of the proline-rich region (P2: residues S198-Q244) with one domain of the pseudo-repeat domain (R2: residues V275-S305; or R3: residues V306-V336) and thus represent two important sequence properties of the full-length htau40 protein (Fig. 4A). To avoid formation of intermolecular dimers, the native cysteine residues, i.e. C291 (R2) and C322 (R3), were substituted by alanine. This mutation was also reported to improve the quality of ssNMR spectra^13^. The peptides were produced recombinantly in *E. coli* (Supplementary Fig. S4).

The intrinsically disordered properties of P2R2 and P2R3 were further investigated using Circular Dichroism. CD spectra of P2R2 and P2R3 displayed a minimum at $\sim \, 200 \, \text {nm}$, indicative for random coil conformation (Fig. 4B and Supplementary Table S2). The 2D $^{1}\text {H}$-$^{15}\text {N}$ HSQC spectra, acquired on either P2R2 or P2R3 displayed low chemical shift dispersion, ranging from $\sim$ 8.0 to $\sim$ 8.8 ppm (Fig. 4C). The backbone resonance assignment of P2R2 and P2R3 was obtained by measurement of 3D NMR experiments and was further validated through comparison with the backbone resonance assignment of htau40^25^. The calculation of the C$\alpha$ chemical shift difference ($\Delta$C$\alpha$) between htau40 and either P2R2 or P2R3 (Fig. 4D) showed differences $< \sim \, 0.4 \, \text {ppm}$, with the exception for the region surrounding the substitutions C291A (P2R2) and C322A (P2R3), respectively, and the residues at the C- and N-terminus of each sequence. Thus, the analysis demonstrated that the local conformational properties within each of the domains (P2, R2, R3) of the two peptides in solution are similar to those of full-length tau.

### Distinct fibril morphology from different pseudo-repeats

**Figure 5:**
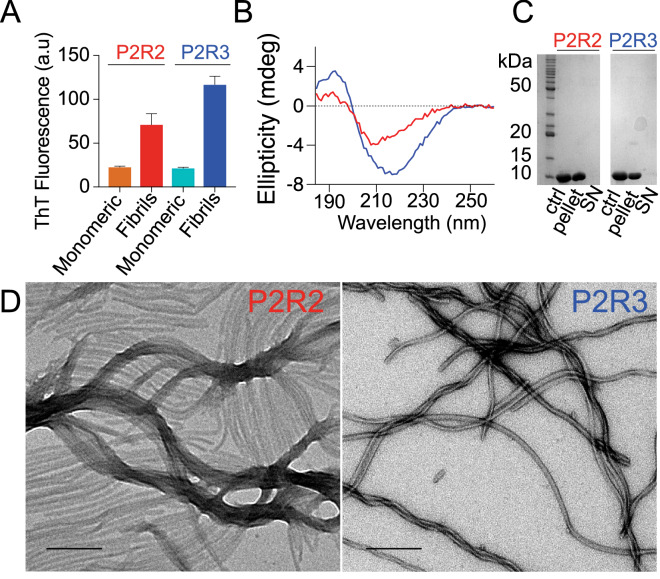
Amyloid fibrils of P2R2 and P2R3 have distinct morphologies. (**A**) ThT fluorescence intensities of P2R2 (red) and P2R3 (blue) fibrils. Data for the peptides in the monomeric form are also shown: P2R2 (orange) and P2R3 (cyan). Error bars indicate the standard deviation over three sample replicates. (**B**) CD spectra of in vitro fibrils of P2R2 (red) and P2R3 (blue) displaying minimuma at 210 nm and 210 nm, indicative of $\beta$-sheet conformation. (**C**) SDS-PAGE gel of the fibrils of P2R2 and P2R3 after centrifugation: ctrl, negative control containing only P2R2 or P2R3 in the monomeric form; SN, supernatant removed after centrifugation). The full-length gel is presented in Supplementary Figure S8. (**D**) Electron micrographs of P2R2 (left) and P2R3 (right) fibrils. Scale bars 500 nm.

To convert the soluble peptides into fibrils, heparin was added to $200 \, \upmu \text {M}$ P2R2 (or P2R3) in 25 mM Tris-HCl buffer at pH 7.4 to a final concentration of $50 \, \upmu \text {M}$ (1:4 heparin:peptide ratio)^20^. Similar to K32, ThT fluorescence measurements were used to monitor the fibrillization reaction (Fig. 5A). Fibrils of P2R2 and P2R3 displayed differences in the ThT fluorescence intensities, suggesting either that the P2R2 peptide had a decreased efficiency in the fibrils formation or that its fibrils interacted differently with the amyloid dye. The amount of protein which aggregated into fibrils was similar for the two peptides, as shown by the picture of the SDS-PAGE gel of the pellet and the supernatant fractions (Fig. 5C; the full-length gel is presented in Supplementary Figure S8). This observation indicated that the weaker ThT intensity observed in case of P2R2 was not caused by inefficient fibrillization. Because the intensity of ThT fluorescence is influenced by the conformation of the fibrils with which it interacts^52^, it rather suggested that the fibrils of P2R2 have a different conformation when compared to P2R3 fibrils. After resuspension in deionized water, the pellet was used for CD measurements. The resulting spectra showed minima indicative for $\beta$-sheet structure, $\sim \, 220 \, \text {nm}$ and $\sim \, 210 \, \text {nm}$ for P2R3 and for P2R2 respectively. However the minimum was more pronounced in the spectrum of P2R3 (Fig. 5B and Supplementary Table S2). Consistent with the ThT fluorescence data, the CD spectra pointed to a difference in the conformation of P2R2 and P2R3 fibrils. This was further supported by electron microscopy performed on the fibrils: a more twisted morphology was observed for P2R3 fibrils, while P2R2 fibrils were mostly straight (Fig. 5D).

### The rigid core of P2R2 and P2R3 fibrils

**Figure 6:**
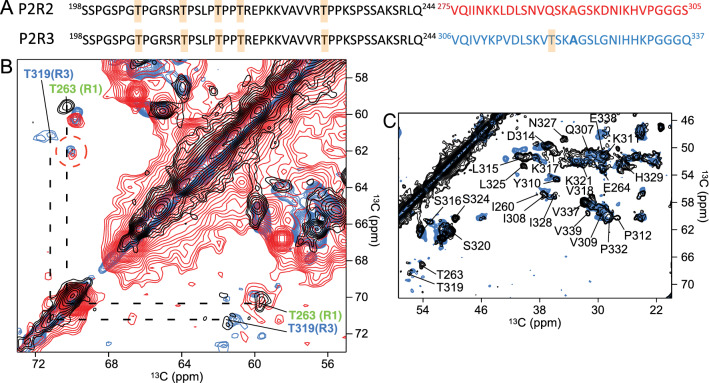
Threonine resonance region in the PDSD spectra of P2R2 and P2R3. (**A**) Amino acid sequences of P2R2 and P2R3; the amino acids belonging to the R2 and the R3 domains are indicated in red and blue, respectively. The threonine residues present in the sequences are highlighted in orange boxes. (**B**) Superposition of the threonine C$\alpha$/C$\beta$ region of the spectra of K19 (black), P2R2 (red) and P2R3 fibrils. Threonine residues belonging to the R1 and the R3 domain are indicated in green and blue, respectively. Signals arising in this region in the spectra of P2R2 (red) and P2R3 (blue) suggest that residues in P2 are rigidified upon fibrillization of P2R2/P2R3. (**C**) Superposition of the PDSD spectra of fibrils of K19 (black, Xiang et al.^40^) and P2R3 (blue). The assignment of K19 (black) reported by Xiang et al.^40^ is shown on top of the PDSD spectrum of K19.

Following a similar approach as for K32, 2D PDSD experiments were performed on uniformly $^{15}\text {N}/^{13}\text {C}$-labeled P2R2 and P2R3 fibrils. Because the amount of fibrils was higher when compared to K32, a 3.2 mm rotor was used for the measurements. The resulting spectra displayed good resolution and the cross peaks were more isolated, most likely because of the decreased number of residues in the peptides when compared to K32 (Supplementary Fig. S5A). In the spectra of both peptides, signals appeared in the region corresponding to the C$\alpha$/C$\beta$ resonances of threonine residues. Moreover, two of the P2R2 cross peaks observed in the threonine C$\alpha$/C$\beta$ region above the diagonal superimpose with cross peaks of P2R3 fibrils, suggesting that the overlapping cross peaks originate from the same two threonine residues (Supplementary Fig. S5A and Fig. 6B). Notably, only one threonine residue is present in the R3 domain, i.e. T319, while the R2 domain contains none (Fig. 6A). Thus, the two superimposing cross peaks in the PDSD spectra of P2R2 and P2R3 respectively should originate from the P2 domain, which contains five threonine residues. Given the presence of the R3 domain in both P2R3 and K19, the PDSD spectrum of K19 fibrils was then superimposed onto that of P2R3. The comparison of the PDSD spectra of P2R3 fibrils and K19 fibrils demonstrated that many cross peaks superimposed. Based on this superposition, a number of P2R3 resonances were tentatively assigned (Fig. 6C). This included the only aromatic residue, Y310. In addition, the cross peak of T319 overlaid with a cross peak in the threonine C$\alpha$/C$\beta$ region of P2R3 fibrils, which was not observed in P2R2 fibrils (Fig. 6B). This further supports the assignment of the two threonine cross peaks, which are observed in P2R2 fibrils (Fig. 6B), to the P2 domain. In the PDSD spectrum of P2R2, a few cross peaks of the R2 domain were tentatively assigned using a recently reported resonance assignment of htau40 fibrils (Fig. S5B)^27^. The comparison suggested that the R2 domain is part of the rigid cross-$\beta$ structure in P2R2 fibrils, in agreement with the cryo-EM structure of the cross-$\beta$ core of heparin-induced tau fibrils^42^. On the contrary, in the cryo-EM structure of the amyloid fibrils purified from the brain of a patient with AD, the R2 domain was excluded from the rigid core^31^. Taken together the data indicate that part of the proline-rich region P2 contributes to the rigid cross-$\beta$ structure core of amyloid fibrils.

### Identification of immobile residues in the proline-rich region P2

**Figure 7:**
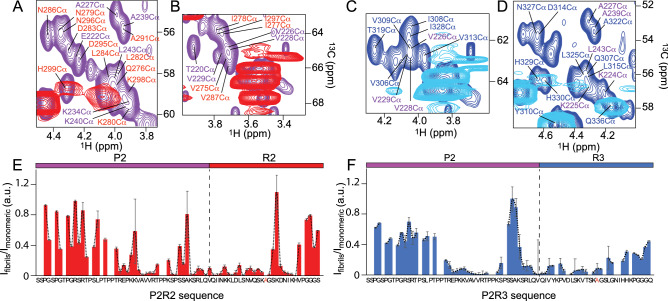
Residue-specific mobility in P2R2 and P2R3 fibrils. (**A**,**B**) Superposition of the $^{1}\text {H}$-$^{13}\text {C}$ HSQC (purple) and the ssNMR INEPT (red) spectra of monomeric and fibrillized P2R2, respectively. Selected regions of the spectra are shown, corresponding to the regions in the P2 and the R2 domains experiencing loss of NMR signal intensity in the INEPT spectrum of the fibrils. (**C**,**D**) Superposition of the $^{1}\text {H}$-$^{13}\text {C}$ HSQC (blue) and the ssNMR INEPT (cyan) spectra of monomeric and fibrillized P2R3, respectively. (**E**) NMR signal intensity ratios between fibrillized and monomeric P2R2. $\textit{I}_{{\text {fibrils}}}$ and $\textit{I}_{{\text {monomeric}}}$ correspond to the are cross peak intensities of the fibrils in the INEPT and the monomeric protein in the $^{1}\text {H}$-$^{13}\text {C}$ HSQC spectra, respectively. Error bars were calculated on the basis of signal-to-noise ratios in the spectra. The domain organization of P2R2 is represented on top. The dashed line shows a three-residue average. (**F**) NMR signal intensity ratios between the fibrillized and the monomeric forms of P2R3. $\textit{I}_{{\text {fibrils}}}$ and $\textit{I}_{{\text {monomeric}}}$ are cross peak intensities of the fibrils in the ssNMR INEPT spectrum and the monomeric protein in the $^{1}\text {H}$-$^{13}\text {C}$ HSQC spectrum, respectively.

The flexible regions of P2R2 and P2R3 fibrils were probed by measuring ssNMR 2D INEPT-based $^{1}\text {H}$-$^{13} \text {C}$ through-bond correlation experiments. In the resulting spectra, several cross peaks were observed for both P2R2 and P2R3 fibrils (Supplementary Fig. S6), as well as an overall similarity, which suggested that similar regions remain flexible in P2R2 and P2R3 fibrils. Using the strategy described above for K32, the INEPT spectra of P2R2 and P2R3 fibrils were compared to the respective $^{1}\text {H}$-$^{13} \text {C}$ HSQC spectra of the monomeric peptides (Fig. 7 and Supplementary Fig. S6). In contrast to the spectra of K32, the C$\alpha$ and C$\beta$ resonances in the $^{1}\text {H}$-$^{13}\text {C}$ HSQC spectra of P2R2 and P2R3 fibrils were more dispersed. This simplified their analysis and showed that resonances from proline, glycine and serine residues are present in the 2D INEPT spectra of P2R2 and P2R3 fibrils.

A number of cross peaks were only present in the $^{1}\text {H}$-$^{13}\text {C}$ HSQC spectra of the monomeric peptides, but were not observed in the ssNMR INEPT spectra. A detailed analysis of the intensities of C$\alpha$ resonances in the fibrillated $\left( \textit{I}_{{\text {fibrils}}}\right)$ and monomeric $\left( \textit{I}_{{\text {monomeric}}}\right)$ spectra identified the residues, which remain flexible upon aggregation into amyloid fibrils (Fig. 7E,F). The residue-specific analysis of P2R3 demonstrated that several residues from the R3 domain including V306, I308, V309, D314, N327 and I328 were not observed in the 2D ssNMR INEPT spectrum (Fig. 7C,D, blue for the amino acids in R3). In addition, K224, V226, A227, V228 and V229, which are part of the $^{225}{\text {KVAVVRT}}^{231}$ sequence in the P2 region were broadened beyond detection in the 2D ssNMR INEPT spectrum of P2R3 fibrils (Fig. 7C,D, purple for the amino acids in P2). The intensity plot obtained by the analysis of the spectra, indicates the region which experience signal broadening or completely disappear Fig. 7F).

In the case of P2R2, the cross peaks of the R2 residues I278, D283 and I297 were severely attenuated when compared to the HSQC of the monomeric peptide (Fig. 7A,B, red for the amino acids in R2). In addition, the cross peaks of the residues T220, R221, E222 and R230, which are flanking the hydrophobic stretch $^{225}{\text {KVAVVRT}}^{231}$ in P2, were strongly attenuated (Fig. 7A,B, purple for the amino acids in P2). The residues V226, A227, V228 and V229, located in the $^{225}{\text {KVAVVRT}}^{231}$ sequence, were completely missing from the 2D ssNMR INEPT spectrum of P2R2 fibrils (Fig. 7A,B, purple for the amino acids in P2). Similarly, the intensity plot obtained by the analysis of the spectra of P2R2, highlights the regions which experience signal broadening or completely disappear Fig. 7E). The analysis further supports a contribution of the P2 domain to the cross-$\beta$ core of amyloid fibrils of P2R2 and P2R3, consistent with the observation of cross peaks in the 2D PDSD spectra of these fibrils that can only be attributed to residues of P2 (Fig. 6 and Fig. S5A). The linker between the $\beta$-strand in R2 (or R3) and the rigid residues in P2 (at least residues $^{225}{\text {KVAVVRT}}^{231}$) comprises residues $^{236}{\text {PSS}}^{238}$ (Fig. 7E,F). Assuming that all residues that are broadened beyond detection form $\beta$-structure, the analysis suggests the presence of two $\beta$-strands in P2R3 (one in P2 and one in R3). In the case of P2R2, an additional short $\beta$-strand might be present at the C-terminus of R2 $\left( ^{296}{\text {NIK}}^{298}\right)$ (Fig. 7E).

### Comparison of the PDSD spectra of P2R2, P2R3 and K32

The P2R2 and P2R3 polypeptides proved to be useful models to probe the contribution of the P2 domain to the cross-$\beta$ structure of heparin-induced tau fibrils. Especially, the smaller number of residues facilitated a residue-specific analysis of the ssNMR spectra. The comparative analysis of the PDSD spectra of K19, P2R2 and P2R3 suggested that the signals arising in the C$\alpha$/C$\beta$ threonine region of the spectra belong to the P2 domain. Then, the K32 PDSD spectrum was superimposed onto the PDSD spectra of P2R2 and P2R3 (Supplementary Figure S7). As shown in the superposition of the spectra, the cross peaks, which had been assigned to the threonine residues in the P2 domain of P2R2 and P2R3, partially matched with cross peaks in the K32 spectrum. This suggested—although the severe signal broadening in the K32 spectrum made further analysis challenging—that the P2 domain might also be contributing to the cross-$\beta$ structure of K32 fibrils.

## Discussion

The proline-rich region of tau is linked to the protein’s role in neurodegenerative diseases^53^. Phosphorylation of the proline-rich region can be detected by several antibodies and is associated with neurodegeneration^44,54,55^. In addition, immunohistochemistry and X-ray crystallographic studies reported that antibodies present in the blood of healthy individuals recognize epitopes in the P2 domain^56,57^. Despite its importance for tau pathology, however, the proline-rich region was not observed in cryo-EM structures of tau fibrils purified from the brains of patients with AD and PiD^30,31^. In addition, the proline-rich region was missing in the cryo-EM structures of amyloid fibrils of htau40 aggregated in vitro in the presence of heparin^42^. The structural role of the proline-rich region in amyloid fibrils of tau is therefore unknown. To gain insight into the structural properties of the proline-rich region of tau in amyloid fibrils, we selected the construct K32, because it contains the P2 domain of the proline-rich region, the four imperfect repeats and the downstream region ${\text {R}}^\prime$ of htau40^7^. Fibrils of K32 were obtained upon addition of heparin and subsequently characterized using different biophysical approaches (Fig. 1). Quantification of the secondary structure content^58^ detected by CD resulted in the following distribution: $\sim 8\% \, \alpha {\text {-helix}}$, $\sim 63\% \, \beta {\text {-sheet}}$, and $\sim 28\%$ turn-like/random-coil conformation (Supplementary Table S2). Because the sequence of K32 comprises 194 residues, the CD analysis suggested that more than 100 residues fold into cross-$\beta$ structure upon aggregation into amyloid fibrils. In the cryo-EM structure of amyloid fibrils of htau40 aggregated in the presence of heparin, i.e. in similar conditions as used here for K32, residues from the C-terminus of R1 and all the residues located in R2 (31 residues) and R3 (31 residues) folded into rigid cross-$\beta$ structure.

In order to gain further insight into the structure of K32 fibrils, ssNMR experiments were performed. The superposition of the PDSD spectra of K32 and K19 suggested that residues belonging to the R1 and R3 domains are part of the rigid cross-$\beta$ structure of amyloid fibrils formed by both of these tau constructs in vitro and in presence of heparin. Severe signal overlap in the spectra of K32 precluded a more detailed analysis. At the current stage, it therefore remains unknown if the tau chain folds into a similar conformation in amyloid fibrils of K32 and K19. Because K19 lacks repeat R2 and thus can be regarded as a model for amyloid fibrils formed by three-repeat isoforms of tau, it indeed is more likely that K19 and K32, which contains four imperfect repeats display a different amyloid fold. Consistent with this hypothesis, the cryo-EM structures of amyloid fibrils purified from patients with AD, in which both three-repeat and four-repeat isoforms are detected in insoluble protein deposits^31^, differ from the cryo-EM structure of amyloid fibrils purified from PiD^30^ i.e. a disease that is characterized by the pathological aggregation of three-repeat isoforms of tau^59^. A further factor influencing the structural properties of tau fibrils, is the oxidation of tau’s two native cysteine residues C291 and C322. These cysteine residues are located in the R2 and R3 domain, respectively, and can form inter- and intra-molecular disulfide bridges in oxidizing conditions^60^.

In addition, the analysis of ssNMR 2D INEPT-based $^{1}\text {H}$-$^{13}\text {C}$ through-bond correlation experiments showed that several proline, glycine and serine residues maintained high flexibility in K32 fibrils. Residues belonging to the R3 domain, which have been reported to be part of the rigid core of the fibrils^30,31^, the R2 domain, observed in the core of heparin-induced fibrils^42^, as well as residues from the P2 domain, e.g. V226, A227, V229 and A239, experienced a decrease in their intensity (Fig. 3). For several isolated resonances in the C$\beta$ region of the spectrum also a complete loss of signal intensity was observed (Fig. 3). The missing peaks belonged to residues located in the R1, the R3 and the R4 pseudo-repeats. The analysis suggested that the R1 and the R3 domain are involved in the formation of the rigid core of K32 fibrils. Some of the signal broadening could also be caused by intermediate exchange. Because of the partial loss of signal intensity observed for the residues in the P2 domain, a partial rigidification of P2 in amyloid fibrils of K32 is likely.

**Figure 8:**
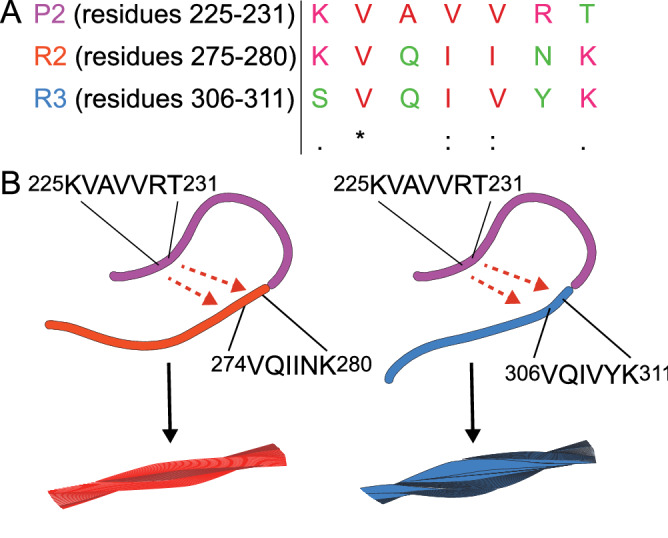
Recruitment of the P2 domain to the core of P2R2 and P2R3 fibrils. Sequence alignment of the hydrophobic sequences in P2, R2 and R3. Symbol legend: “*” indicates conserved residues, “:” indicates high similarity and "$\cdot$" indicates weak similarity between aminoacids. The colors in the table refer to hydrophobic residues (red), basic aminoacids (magenta) and amino acid residues with hydroxyl group and/or an amine group (green). (**B**) Schematic representation of the recruitment of the P2 domain into tau fibrils, via establishment of hydrophobic interactions.

Further support for a rigidification of part of the proline-rich region P2 upon tau fibrillization was provided by solid-state NMR experiments of the model polypeptides P2R2 and P2R3: resonances of residues $^{225}{\text {KVAVVRT}}^{231}$ of the P2 domain were strongly attenuated when compared to the monomeric state of the peptides (Fig.7E,F). The $^{225}{\text {KVAVVRT}}^{231}$ sequence is rich in hydrophobic residues and is homologue to the hexapeptides in repeats R2 and R3 (Fig. 8A)^11^. The three sequences $^{225}{\text {KVAVVRT}}^{231}$, $^{275}{\text {VQIINK}}^{280}$ and $^{306}{\text {VQIVYK}}^{311}$ share a high number of hydrophobic residues, e.g. valine, alanine and/or isoleucine residues. Thus, the establishment of intramolecular hydrophobic interactions between the $^{225}{\text {KVAVVRT}}^{231}$ sequence in the P2 domain and the hexapeptides $^{275}{\text {VQIINK}}^{280}$ (R2) and $^{306}{\text {VQIVYK}}^{311}$ (R3) could be responsible for the rigidification of the P2 region in tau amyloid fibrils (Fig. 8B). Responsible for the rigidification of the P2 region could also be intermolecular interactions established between the P2 domains or between the P2 domain and the R2/R3 domain of two distinct tau molecules. The latter possibility would explain the formation of fibril bundles, as observed in Fig. 5D.

The partial rigidification of the P2 domain is also in agreement with a recent ssNMR study of heparin-induced fibrils of the four-repeat tau construct 0N4R, in which the proline-rich region was defined as semi-mobile^27^. The current results together with previous studies suggest that the semi-mobility of the proline-rich region P2 might be necessary for the regulation of processes at the beginning or right after the aggregation of tau induced by heparin. This is also supported by the finding that antibodies recognizing epitopes of the P2 region are present in the blood of healthy patients^57^.

Because the P2 region is an important site of post-translational modifications, i.e. disease-associated phosphorylation of serine and threonine residues^61^, phosphorylation of the P2 region is expected to further modify the structural properties of this region and thus contribute to aberrant tau aggregation and neurotoxicity. Of particular relevance might be T231, which is part of the $^{225}{\text {KVAVVRT}}^{231}$ sequence and has been found to be phosphorylated in the CSF of AD patients^62,63^.

## Methods

### Protein purification

The plasmid carrying the coding sequence of the K32 construct, which comprises the P2 domain (residues S198-Q244) and the complete pseudo-repeats region, including repeats R1, R2, R3, R4 and ${\text {R}}^\prime$ (residues Q244-S400) of full-length htau40, was kindly provided by the laboratory of Prof. Dr. Eckhard Mandelkow. Protein expression and purification was performed as previously described^7^. The concentration of K32 (25 kDa) was confirmed using bicinchoninic acid (BCA) assay kit (Sigma-Aldrich, Germany). The plasmids encoding the DNA sequence of either P2R2 or P2R3, which combine the amino acid sequences of the P2 domain (residues S198-Q244) with either the R2 domain (residues V275-S305) or the R3 domain (residues V306-Q336) of htau40, were purchased from GeneArt gene synthesis from Invitrogen, Thermo Fisher Scientific (Germany). The two native cysteine residues C291 and C322 of htau40, located in the R2 and R3 domains respectively, were replaced by alanine in the polypeptides. The DNA sequences of P2R2 and P2R3 were cloned into a pET28a vector, between the BamHI and HindIII restriction sites. Rosetta${}^{\text{TM}}$ 2 (DE3) cells (Novagen, Germany) containing the pET28a plasmid, carrying either the P2R2 or the P2R3 sequence, were grown in M9 medium and induced with 0.5 mM IPTG; cells were harvested after 4 h of protein expression at 37 °C by centrifugation at 7500 rpm, using a JLA-8.1 rotor in a Beckman Coulter Avanti centrifuge, for 30 min. Pellets were resuspended in a 20 mM Tris-HCl lysis buffer, pH 8, containing 500 mM NaCl, Lysozyme, EDTA-free Protease inhibitor mix, DNase, $0.2 \, {\text {mM MgCl}}_2$ and 5 mM DTT. Cell membranes were disrupted by sonification cycles followed by sedimentation of insoluble particles, which was achieved by centrifugation at 20,000 rpm for 30 min in a JA 25.50 in a Beckman Coulter Avanti centrifuge. The purification steps were performed using Qiagen Ni-NTA Agarose for gravity-flow chromatography (Qiagen, Germany), with washing steps with a buffer containing increasing concentration of imidazole. The removal of the His-tag was achieved by overnight TEV cleavage in dialysis at 4 °C. To achieve maximal purity of the sample, P2R2 and P2R3 samples, in a 20 mM Tris-HCl buffer at pH 8 and containing 250 mM NaCl, were loaded on a HiLoad${}^{\text{TM}}$ 16/200 Superdex 75 pg (GE, Healthcare Life Sciences, Germany). Because of the lack of tryptophans, the concentration of P2R2 (7 kDa) and P2R3 (7 kDa) was estimated using a sequence-specific extinction coefficient for absorbance at 205 nm^64^. For P2R2 and P2R3, ${\text {Abs}}_{205}$ was $225,230 \, {\text {M}}^{-1} \, {\text {cm}}^{-1}$ and $235,710 \, {\text {M}}^{-1} \, {\text {cm}}^{-1}$, respectively.

### Circular dichroism of monomeric proteins and fibrils

CD spectra of monomeric K32, P2R2 and P2R3 peptides were acquired on a Chirascan (Applied Photophysics, UK) spectrometer. Samples in sodium phosphate buffer 50 mM, pH 6.8, 50 mM NaCl were diluted in ${\text {ddH}}_{2} \text {O}$ to reach a final protein concentration of $12.5 \, \upmu \text {M}$ for K32 and $100 \, \upmu \text {M}$ for P2R2 and P2R3. Measurements were performed in a 0.1 cm light path quartz cuvette at room temperature using 100 nm/min, a bandwidth of 1.0 nm and 3 repeats per measurement. Fibrils obtained from K32, P2R2 and P2R3 were pelleted by ultracentrifugation at 200,000 *g*, at 37 °C for 1 h in a TLA 100.3 rotor in a bench ultracentrifuge (Beckman Coulter, Optima MAX XP) and resuspended in ${\text {ddH}}_{2} \text {O}$. Circular dichroism spectra were measured in a 0.1 cm light path quartz cuvette at room temperature, using 100 nm/min, a bandwidth of 1.0 nm and 3 repeats per measurement. For all the CD spectra the baseline correction was performed subtracting the spectrum of the buffer acquired with the same parameter settings. Data for the monomeric protein are expressed as mean residue ellipticity (MRE), while for the CD spectra of the fibrils, data are reported in ellipticity units (mdeg). Quantification of secondary structure content was achieved using Dichroweb^58,65^.

### Fibrillization reactions

The fibrillization of unlabeled and $^{15}\text {N}/^{13}\text {C}$-labeled K32, P2R2 and P2R3 samples was induced using heparin salt (Mr 2 × 104 g/mol, Carl Roth, Germany) in a heparin-to-protein molar ratio of 1:4. Either P2R2 or P2R3 ($200 \, \upmu \text {M}$) were incubated with heparin ($50 \, \upmu \text {M}$) in a buffer containing Tris-HCl 25 mM, pH 7.4, 0.02% ${\text {NaN}}_{3}$. In the case of K32, $100 \, \upmu \text {M}$ of final protein concentration were incubated with heparin ($25 \, \upmu \text {M}$) in a buffer containing Tris-HCl 25 mM, pH 7.4, 0.02% ${\text {NaN}}_{3}$ and 2 mM DTT freshly added just before starting the reaction. Samples were incubated for 3 days at 37 °C. During this time, ThT measurements of each sample were performed to monitor the fibrillization process. The reaction was then stopped by ultracentrifugation at 200,000*g*, at 37 °C for 1 h in a TLA 100.3 rotor in a bench ultracentrifuge (Beckman Coulter, Optima MAX XP). The composition of the supernatant and the pellet was monitored by SDS-PAGE.

### ThT fluorescence assays

Fibril formation was monitored performing a Thioflavin T (ThT) fluorescence assay^66^. The measurements were carried on a 96 well plate in a Cary Eclipse fluorescence spectrophotometer at room temperature, using an excitation and an emission wavelengths of 440 nm and 482 nm, respectively. $1 \, \upmu \text {l}$ of protein sample reaction mixture was thoroughly pipetted into $180 \, \upmu \text {l}$ of ThT working solution (glycine 50 mM, pH 8 and $5 \, \upmu \text {M}$ ThT). Experiments were performed in triplicates including a negative control of ThT working solution in each experiment.

### Electron microscopy

The morphology of the fibrils was analyzed by negative-stain transmission electron microscopy (TEM). For each sample the fibrils were harvested by ultracentrifugation, discarding the supernatant followed by resuspension in fresh fibrillization buffer ($50{-}60 \, \upmu \text {l}$). Samples were transferred onto a continuous carbon coated copper grid and buffer removal was achieved using a filter paper. Negative staining was performed by adding 1% (w/v) of aqu. uranyl acetate solution followed by drying process with a filter paper. EM pictures were taken using a FEI CM 120 electron microscope with Tietz F416 CMOS camera.

### NMR spectroscopy

2D $^{1}\text {H}$-$^{13}\text {C}$ and $^{1} \text {H}$-$^{15}\text {N}$ HSQC experiments of $200 \, \upmu \text {M}$ uniformly $^{15} \text {N}/^{13} \text {C}$ labeled K32 were recorded at 5 °C, in 50 mM sodium phosphate buffer, pH 6.8, 10% ${\text {D}}_{2}$0, 0.02% ${\text {NaN}}_{3}$ and 2 mM DTT, added freshly before the measurement. Spectra were acquired on a 700 MHz spectrometer equipped with a cryogenically-cooled triple resonance probe (Bruker, Germany). HNCA, HNCACB and HNCO spectra for backbone assignment, (H)CC(CO)NH- and H(CC)(CO)NH-TOCSY spectra for side chain assignment, were recorded on a 700 MHz spectrometer equipped with a cryogenically-cooled triple resonance probe (Bruker, Germany). For characterization of the monomeric form of the P2R2 and P2R3 tau peptides, 2D $^{1}\text {H}$-$^{13}\text {C}$ and $^{1}\text {H}$-$^{15}\text {N}$ HSQCs were recorded at 5 °C on 1 mM concentrated samples of uniformly $^{15}\text {N}/^{13}\text {C}$ labeled peptides in 50 mM sodium phosphate buffer, pH 6.8, 10% ${\text {D}}_{2} 0$ and 0.02% ${\text {NaN}}_{3}$. Spectra of P2R2 were acquired on a Bruker Avance III HD spectrometers operating at 600 MHz and spectra of P2R3 were acquired on a 600 MHz Bruker spectrometer equipped with a triple-resonance cryogenically-cooled NMR probe. For the sequential backbone assignment of P2R2 and P2R3, a suite of triple-resonance experiments (HNCA, HNCACB, HNCACO, HNCO) was recorded on a 600 MHz Bruker Avance III HD spectrometer equipped with a room temperature triple resonance probe as well as on a 600 MHz Bruker spectrometer equipped with a triple-resonance cryogenically-cooled NMR probe. Experiments for sidechain assignment ((H)CC(CO)NH- and H(CC)(CO)NH-TOCSY) were acquired on a 700 MHz spectrometer equipped with a cryogenically-cooled triple resonance probe (Bruker, Germany). All the spectra were processed using Topspin 3.5pl7 software and assignments were performed with ccpNMR^67^. In addition, a full-length human tau assignment^14^ was used to validate the assignment of each construct.

For the acquisition of ssNMR spectra, $^{15}\text {N}/^{13}\text {C}$-labeled fibrils of P2R2 ($\sim$ 20 mg), P2R3 ($\sim$ 20 mg) and K32 ($\sim$ 12 mg) where ultra-centrifuged in a 45 Ti Ultra Rotor at 127,000*g*, at 37 °C for 1 h (Beckman Coulter, Optima MAX XP) prior to MAS rotor filling and measurement of the ssNMR experiments. DSS (4,4-dimethyl-4-silapentane-1-sulfonic acid) in powder (Sigma-Aldrich) was added to each sample as internal chemical shift reference. PDSD and INEPT experiments were recorded on $^{15}\text {N}/^{13}\text {C}$-labeled K32 fibrils packed into a 1.3 mm rotor. Spectra were acquired at 5 °C and 11 kHz spinning speed on a Bruker 850 MHz wide bore spectrometer (Bruker, Germany, Germany). A mixing time of 20 ms was set to obtain intra-residue correlations. The 2D INEPT transfer-based $^{1}\text {H}$-$^{13}\text {C}$ experiments were performed on the same instrument at 5 °C and 8 kHz. For P2R2 and P2R3, fibrils of uniformly $^{15}\text {N}/^{13}\text {C}$-labeled peptides were packed into a 3.2 mm rotor. PDSD and INEPT spectra were recorded at 5 °C and a spinning speed of 12.5 kHz and 8 kHz, respectively, on a Bruker 850 MHz wide bore spectrometer (Bruker, Germany). A mixing time of 20 ms was set to obtain intra-residue correlations in the PDSD spectra. The parameters referring to the ssNMR experiments are reported in the table S1. Spectra were processed using TopSpin 3.5 pl 7 (Bruker, Germany) and analyzed with Sparky^68^ and ccpNMR^67^.

## Supplementary information

Supplementary Information 1.

## Data Availability

The data generated and/or analysed for this study are included in this published article (and its Supplementary Information files available online)
