# Supplementary material for: Solid-state NMR investigation of the involvement of the P2 region in tau amyloid fibrils
Source: Sci Rep. 2020 Dec 3;10:21210. doi: 10.1038/s41598-020-78161-0 (PMC7712923; doi:10.1038/s41598-020-78161-0)
Supplement: Supplementary file 1 — Supplementary Information 1. [file 41598_2020_78161_MOESM1_ESM.pdf]

Supporting Information
Solid-state NMR investigation of the involvement of the P2 region in tau amyloid fibril

A. Savastano[1], G. Jaipuria[1], L. Andreas[2], E. Mandelkow[3,4], and M. Zweckstetter[1,2,*]

[1]German Center for Neurodegenerative Diseases (DZNE), Von-Siebold-Str. 3a, 37075 Göttingen, Germany.
[2]Max Planck Institute for Biophysical Chemistry, Am Faßberg 11, 37077 Göttingen, Germany.
[3]German Center for Neurodegenerative Diseases (DZNE), Venusberg-Campus 1, 53127 Bonn, Germany.
[4]CAESAR Research Center, Ludwig-Erhard-Allee 2, 53175 Bonn, Germany.
[*]Correspondence: markus.zweckstetter@dzne.de

Supplementary Table S1. List of solution state 3D experiments recorded on K32, P2R2 and P2R3.

Protein	Type of experiment	Spectrometer (MHz)	Pulse sequence	TD[a]
P2R2/P2R3	HNCA	600	hncagp3d	2048 (^{1}H), 48 (^{13}C), 128 (^{15}N)
	HNCO	600	hncogp3d	2048 (^{1}H), 48 (^{13}C), 128 (^{15}N)
	HNCACB	600	hncacbgp3d	2048 (^{1}H), 48 (^{13}C), 128 (^{15}N)
	HN(CO)CACB	600	hncocacbgp3d	2048 (^{1}H), 50 (^{13}C), 114 (^{15}N)
	H(CC)(CO)NH-TOCSY	700	hccconhgp3d2	2048 (^{1}H), 64 (^{13}C), 176 (^{15}N)
	(H)CC(CO)NH-TOCSY	700	hccconhgp3d3	2048 (^{1}H), 40 (^{13}C), 128 (^{15}N)
K32	HNCA	700	hncagp3d	2048 (^{1}H), 64 (^{13}C), 128 (^{15}N)
	HNCO	700	hncogp3d	2048 (^{1}H), 64 (^{13}C), 200 (^{15}N)
	HNCACB	700	hncacbgp3d	2048 (^{1}H), 64 (^{13}C), 200 (^{15}N)
	H(CC)(CO)NH-TOCSY	700	hccconhgp3d2	2048 (^{1}H), 64 (^{13}C), 200 (^{15}N)
	(H)CC(CO)NH-TOCSY	700	hccconhgp3d3	2048 (^{1}H), 80 (^{13}C), 136 (^{15}N)

[a] Time domain.

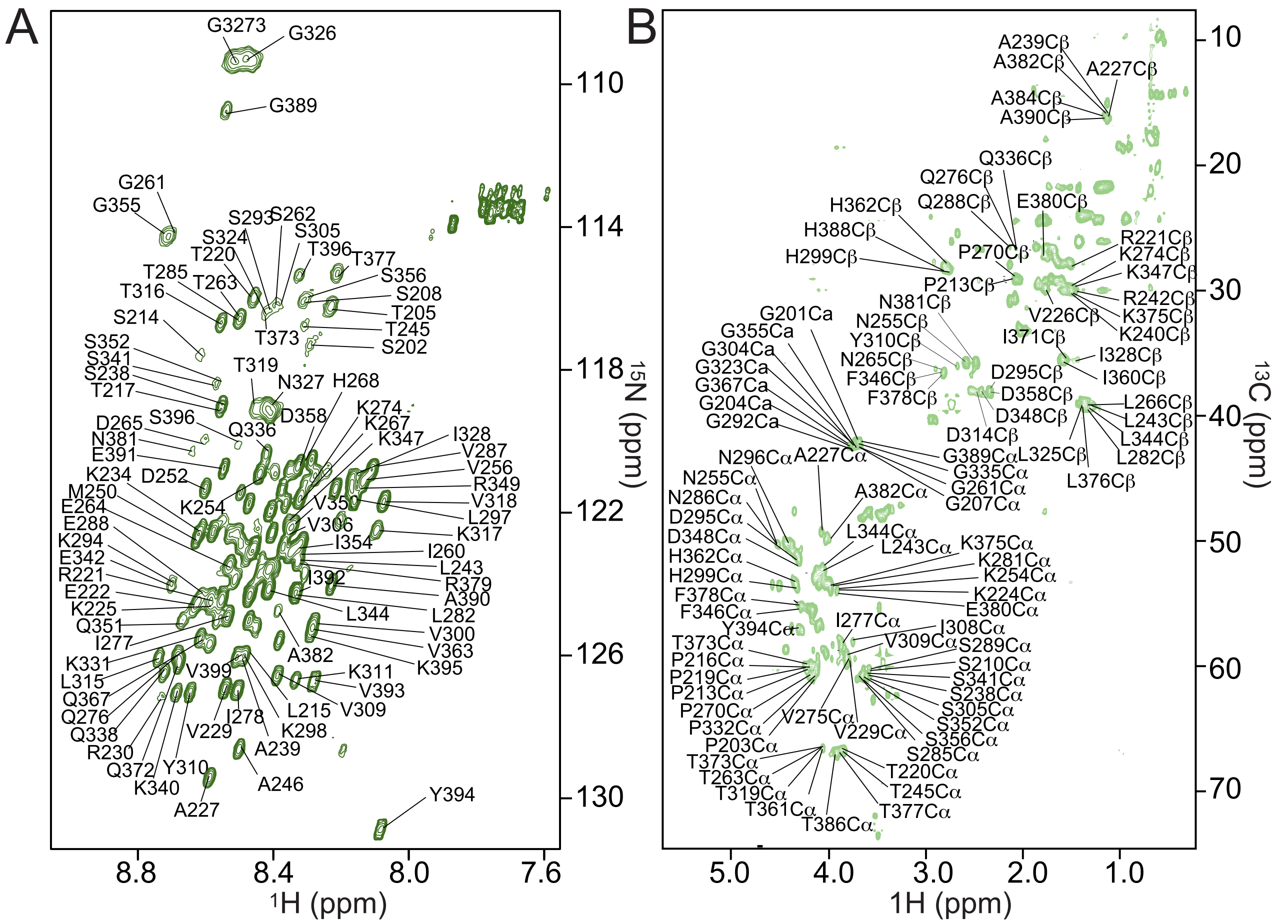

Supplementary Fig. S1. Backbone and side chain assignment of K32 in solution. A) ^{1}H-^{15}N HSQC spectrum recorded on the K32 construct at 5 °C in 50 mM sodium phosphate buffer (2 mM DTT, 0.02 % NaN_3) at pH 6.8. B) ^{1}H-^{13}C HSQC spectrum of K32 measured at 5 °C in 50 mM sodium phosphate buffer (2 mM DTT, 0.02 % NaN_3) at pH 6.8.

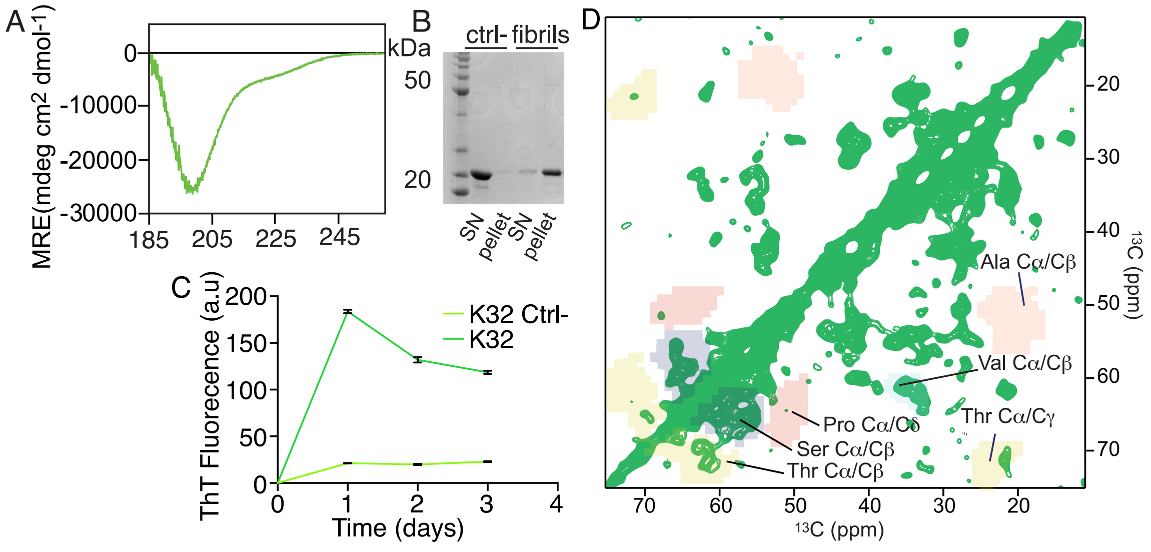

Supplementary Fig. S2. *in vitro* Heparin-induced fibrils of K32. A) CD spectrum of K32 in monomeric form, showing a minimum at 190 nm, typical for random coil secondary structure, also confirmed by quantification by Dichroweb. B) K32 Fibrils formation induced by the addition of heparin. The lanes indicate the negative control, i.e. K32 incubated in absence of heparin, supernatant and pellet, and the supernatant and the pellet for fibrillization reaction; the full-length gel is represented in Fig.S8. C) ThT fluorescense of K32 fibrils over time. D) Regions corresponding to ^{13}C chemical shifts values for selected amino acids [1] are indicated on the spectrum in different colors.

Supplementary Table S2. Secondary structure content calculated using the Dichroweb software on the bases of experimental CD spectra.

	Helix	Strand	Turn	Unordered	Total
P2R2 monomeric	0.07	0.40	0.19	0.33	0.99
P2R3 monomeric	0.07	0.44	0.19	0.32	0.99
K32 monomeric	0.07	0.43	0.19	0.33	0.99
P2R2 fibrils	0.01	0.60	0.27	0.13	0.99
P2R3 fibrils	0.01	0.60	0.27	0.13	0.99
K32 fibrils	0.08	0.63	0.28	0.003	0.99

Supplementary Table S3. Selected parameters for ssNMR experiments performed on tau fibrils

Protein	Type of experiment	Rotor	SW[a]	NS[b]	TD[c]	Spinning Speed
P2R2/P2R3	PDSD	3.2 mm	299 (^{13}C), 299 (^{13}C)	48	1274(^{13}C),1274(^{13}C)	11 KHz
	INEPT	3.2 mm	199 (^{1}H), 14 (^{13}C)	160	2048(^{1}H), 204(^{13}C)	8 KHz
K32	PDSD	1.3 mm	299 (^{13}C), 299 (^{13}C)	64	1274(^{13}C),1274(^{13}C)	12.5 KHz
	INEPT	1.3 mm	199 (^{1}H), 14 (^{13}C)	204	2048(^{1}H), 204(^{13}C)	8 KHz

[a] Spectral Width.
[b] Number of scans.
[c] Time domain.

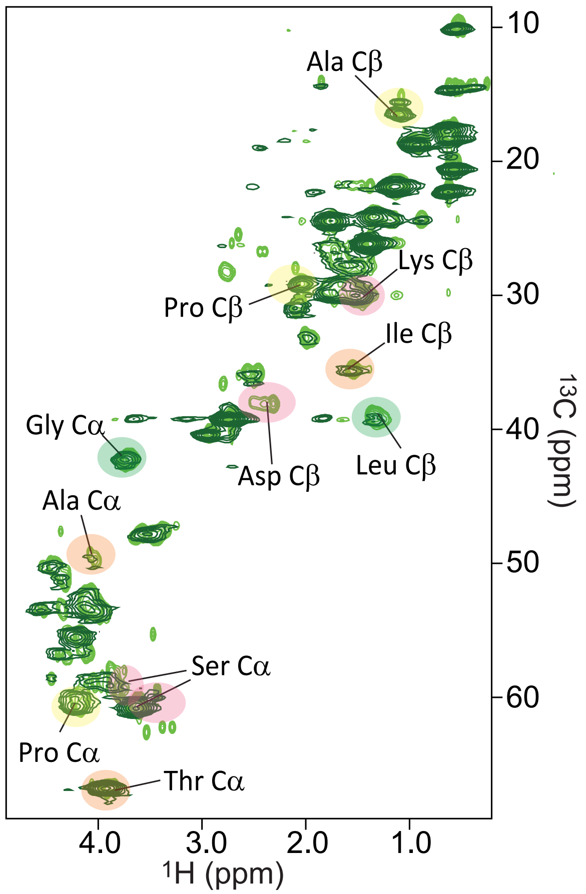

Supplementary Fig. S3. Detection of the flexible regions in K32 fibrils. Superposition of the ^{1}H-^{13}C HSQC spectrum (light green) and the ssNMR INEPT spectrum (dark green) of monomeric and fibrillized K32, respectively. Regions adopting a β-sheet conformation as a result of the fibrils formation cannot be detected in the INEPT-based ssNMR experiment. Regions for the residue type assignments are marked in different colours and the residue and atom type are indicated.

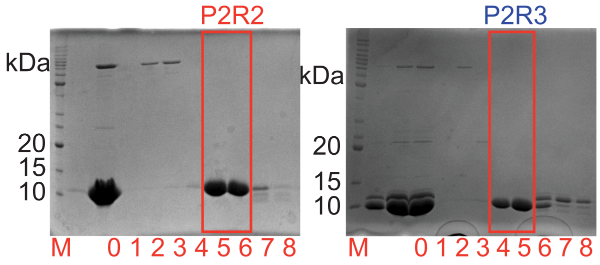

Supplementary Fig. S4. Purification of P2R2 and P2R3 polypeptides by size exclusion chromatography. SDS-PAGE gels of P2R2 and P2R3 size exclusion chromatography fractions. Indicated in the red boxes are the fractions with the highest purity, which were selected. Lane M, protein marker (Benchmark TM); lane "0", sample before the size exclusion chromatography.

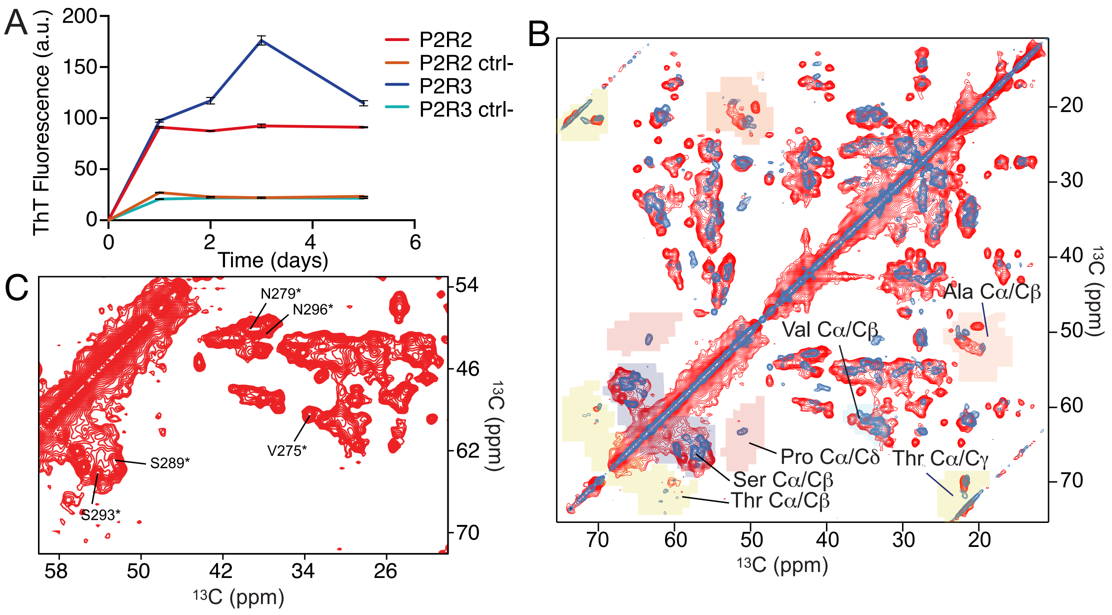

Supplementary Fig. S5. *in vitro* fibrils of P2R2 and P2R3 . A) ThT fluorescensce of K32 fibrils over time. B) Superposition of the PDSD spectra of *in vitro* fibrils obtained from P2R2 (red) and P2R3 (blue). The ^{13}C chemical shifts values of the different amino acid types in β-sheet conformation reported by Fritzsching et al., J Biomol NMR 2013 are indicated in different colors. C) PDSD spectrum of *in vitro* fibrils of P2R2. Indicated are selected resonance assignments reported for the heparin-induced fibrils of 0N4R [2].

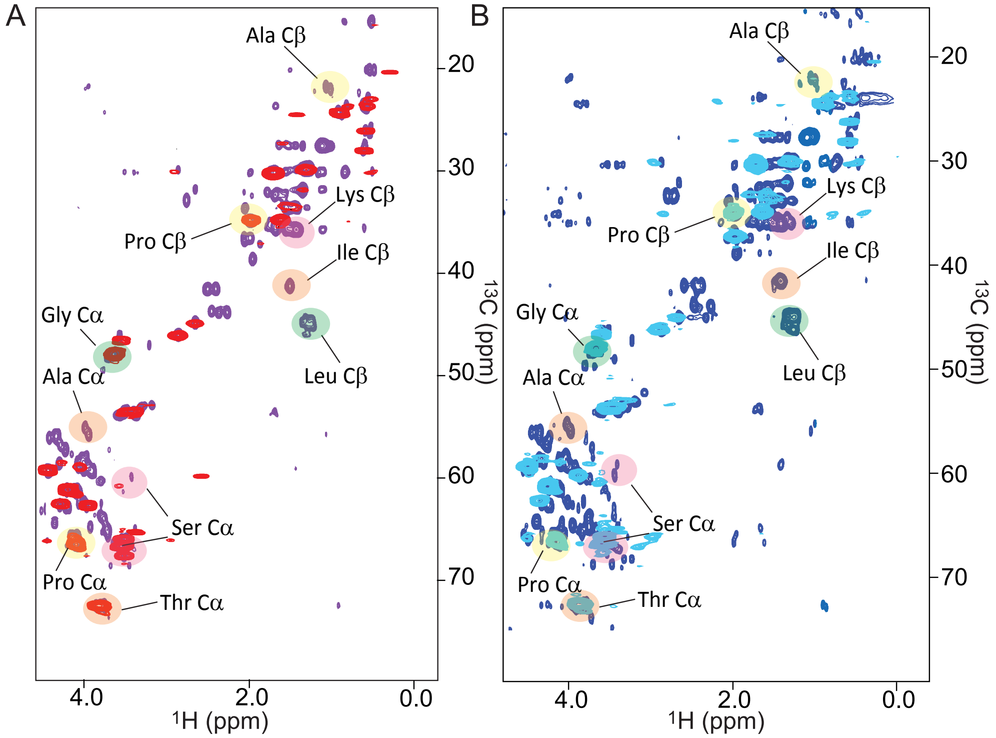

Supplementary Fig. S6. Detection of the flexible regions in P2R2 and P2R3 fibrils. A) Superposition of the ^{1}H-^{13}C HSQC (purple) and the ssNMR INEPT (red) spectra of monomeric and fibrillized P2R2, respectively. Regions adopting β-sheet conformation as a result of fibril formation cannot be detected in the INEPT-based ssNMR experiment. B) Superposition of the ^{1}H-^{13}C HSQC (lightblue) and the ssNMR INEPT (darkblue) spectra of monomeric and fibrillized P2R3, respectively. Regions for the residue type assignments are marked in different colours and the residue and atom type are indicated.

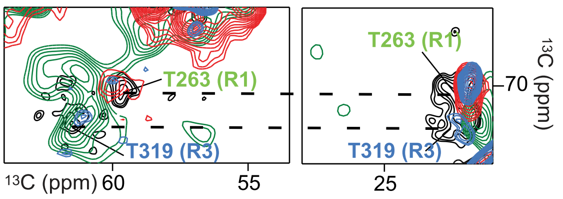

Supplementary Fig. S7. Comparison of threonine signals in the PDSD spectrum of K32, P2R2 and P2R3 fibrils. Cβ/Cγ region of the spectra of K32 (green), K19 (black), P2R2 (red) and P2R3 (blue) fibrils: the comparison shows that also in the spectrum of K32 fibrils peaks arising in the threonine region can be tentatively assigned to T263 and T319, in R1 and R3, respectively.

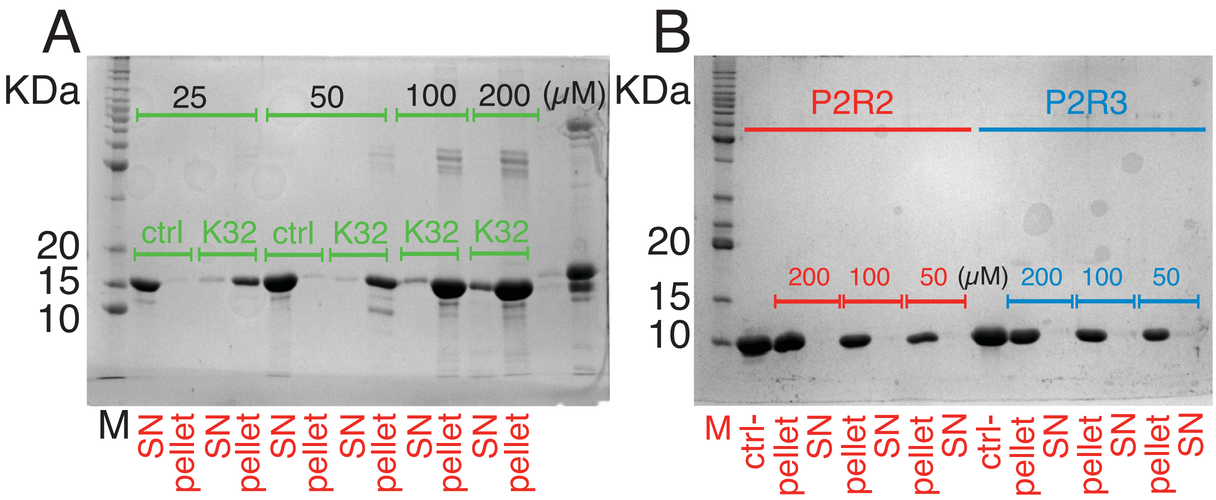

Supplementary Fig. S8. Full-length gels of fibrillization tests for K32, P2R2 and P2R3 A) SDS-PAGE of K32 fibrillization tests at different concentrations. Lane M, protein marker (Benchmark TM); lane "SN", supernatant collected after ultracentrifugation; lane "pellet", pellet resuspended in the same volume as the supernatant (to avoid dilution artifacts) after ultracentrifugation; "ctrl" indicates K32 in the monomeric form, incubated in absence of heparin. The concentrations of K32 in μM, which were tested are indicated on top of the gel. B) SDS-PAGE of P2R2 (red) and P2R3 (blue) fibrillization tests at different concentrations. Lane M, protein marker (Benchmark TM); lane "SN", supernatant collected after ultracentrifugation; lane "pellet", pellet resuspended in the same volume as the supernatant (to avoid dilution artifacts) after ultracentrifugation; "ctrl" indicates P2R2/P2R3 in the monomeric form, incubated in absence of heparin. The concentrations of the polypeptides in μM, which were tested are indicated on top of the gel.

SUPPLEMENTARY REFERENCES

1. Fritzsching, K. J., Y. Yang, K. Schmidt-Rohr, and M. Hong, 2013. Practical use of chemical shift databases for protein solid-state NMR: 2D chemical shift maps and amino-acid assignment with secondary-structure information. *J Biomol NMR* 56:155–67.

2. Dregni, A. J., V. S. Mandala, H. Wu, M. R. Elkins, H. K. Wang, I. Hung, W. F. DeGrado, and M. Hong, 2019. In vitro 0N4R tau fibrils contain a monomorphic beta-sheet core enclosed by dynamically heterogeneous fuzzy coat segments. *Proc Natl Acad Sci U S A* 116:16357–16366.
